# Dietary Inclusion of a *Saccharomyces cerevisiae*-Derived Postbiotic Is Associated with Lower *Salmonella enterica* Burden in Broiler Chickens on a Commercial Farm in Honduras

**DOI:** 10.3390/microorganisms10030544

**Published:** 2022-03-01

**Authors:** W. Evan Chaney, S. Ali Naqvi, Manuel Gutierrez, Abel Gernat, Timothy J. Johnson, Derek Petry

**Affiliations:** 1 Diamond V., Cargill Health Technologies, 2525 60th Ave. SW, Cedar Rapids, IA 52404, USA; agernat@diamondv.com (A.G.); tim_johnson@diamondv.com (T.J.J.); dpetry@diamondv.com (D.P.); 2 Engineering and Data Sciences, Cargill, Incorporated, 15407 McGuinty Road West, Wayzata, MN 15407, USA; ali_naqvi@cargill.com; 3 Agrinvet Laboratorio, Barrio Lempira, 8 Calle, 7 y 8 Avenida, San Pedro Sula 21104, Honduras; gruposymmag@gmail.com

**Keywords:** *Salmonella*, postbiotic, poultry, broiler, ceca, preharvest, feed additive, food safety, intervention, Honduras

## Abstract

Postbiotic feed additives may aid foodborne pathogen reduction during poultry rearing. The study objective was to evaluate a postbiotic additive in parallel to an industry control diet and the subsequent associated burden of *Salmonella enterica* on a single, commercial broiler farm in Honduras. Twelve houses were matched and assigned the standard diet (CON) or standard diet plus postbiotic (SCFP). New litter was placed in each house and retained across flock cycles with sampling prior to each chick placement and three consecutive rearing cycles. At ~33–34 days, 25 ceca were collected on-farm from each house, treatment, and cycle. *Salmonella* prevalence in litter for CON (30.6%) and SCFP (27.8%) were equivalent; however, *Salmonella* load within positive samples was lower (*p* = 0.04) for SCFP (3.81 log_10_ MPN/swab) compared to CON (5.53 log10 MPN/swab). Cecal prevalence of *Salmonella* was lower (*p* = 0.0006) in broilers fed SCFP (3.4%) compared to CON (12.2%). *Salmonella* load within positive ceca were numerically reduced (*p* = 0.121) by 1.45 log_10_ MPN/g for SCFP (2.41 log_10_ MPN/g) over CON (3.86 log_10_ MPN/g). Estimated burden was lower (*p* = 0.003) for SCFP flocks (3.80 log_10_ MPN) compared to CON (7.31 log_10_ MPN). These data demonstrate the preharvest intervention potential of postbiotics to reduce *Salmonella enterica* in broiler chickens.

## 1. Introduction

Countries with robust public health surveillance, reporting, and industry regulatory oversight routinely monitor for agents associated with foodborne illness, especially serovars of *Salmonella enterica*. In the United States, foodborne disease associated with 31 known pathogens results in an estimated 9.4 million illnesses annually and, of those, non-typhoidal *Salmonella* are estimated to cause approximately 1.2 million illnesses, 23,000 hospitalizations, and 450 deaths [1]. In a report from the Interagency Food Safety Analytics Collaboration, more than 75% of illnesses reported from 811 outbreaks between 1998 and 2017 were linked to *Salmonella* and were attributable to seven food categories of which chicken products (14.0%) and eggs (7.9%) together constituted the primary source of over 20% of *Salmonella* outbreaks [2]. In contrast, many developing countries lack robust surveillance programs and public health data on the domestic incidence and attribution of foodborne salmonellosis. Nonetheless, the association of poultry products with *Salmonella* and its corresponding public health threat are globally recognized and, therefore, necessitate *Salmonella* control in an increasingly globalized import and export market [3,4,5].

As developing countries seek to increase poultry production capacity to meet increasing domestic consumption trends, additional opportunities to increase export volumes are often met with more stringent regulatory and food safety-based compliance specifications. For instance, while Honduran raw poultry products have yet to be exported to the United States due to endemic Newcastle disease concerns, raw beef products are approved for export. Establishments producing these products are regulated by SENASA, the federal national service of health and food safety. Export approval is based on demonstrated equivalence of SENASA-specified regulations and food safety process controls, including microbiological testing, to criteria established by the United States Department of Agriculture’s Food Safety and Inspection Service, who conducts the Foreign Supplier Audits and Reports [6]. SENASA has recently established *Salmonella* performance standards for livestock and poultry. As in the United States, Honduran poultry establishments have inspectors who collect chicken carcass rinse samples post-chill, utilizing the reference microbiological methods specified in the FSIS’s Microbiology Laboratory Guidebook 4.11 at the Laboratorio Nacional de Residuos (LANAR) [7]. To remain in compliance, weekly samplings may result in no more than five qualitatively positive *Salmonella* detections within a moving 51-week window [6]. To meet these criteria, robust process controls and post-harvest microbial interventions are commonly deployed. As may be evidenced from the establishment of compliance reports in the United States, and a largely unchanging public health burden of salmonellosis attributed to poultry products, postharvest microbial interventions are not fully efficacious despite many years of technology use, development, and regulatory oversight [8,9].

In recent years, qualitative performance standards have come under scrutiny in favor of a more quantitative-based approach to pathogen testing and associated product safety risk [10]. The FSIS’s recent Roadmap to Reducing *Salmonella* includes potential assessment or evaluation of semiquantitative- or quantitative-based methods to inform *Salmonella* risk assessments [11]. Recent risk modeling studies for *Salmonella* in ground turkey have strengthened the argument that contamination load, and not prevalence alone, may be a more impactful metric to utilize [12,13]. Ultimately, this potential shift towards a load-based approach could impact countries seeking export to the United States and other countries. As such, a continual effort to identify technologies capable of further reducing *Salmonella* risk are also increasingly focused on live production. Interventions and products reducing the prevalence and loads of *Salmonella* in live animals during rearing and at harvest are generally assumed to enhance the efficacy of postharvest interventions utilized in downstream processing plants.

In primary production, chick source, biosecurity, farm inputs, and management practices, including sanitation practices, are fundamental to managing *Salmonella* and general pathogen risk [14,15,16]. In poultry, vaccination with live, attenuated strains of *Salmonella* are commonplace and continue to evolve with variable efficacy [17,18]. While vaccination may confer early protection against wild type *Salmonella* colonization, durability of protection may decrease over time, requiring booster administration [18,19,20]. Feed additive solutions that can be continually coadministered throughout the life of the animal while promoting gut health, performance, and protection against pathogen colonization are therefore attractive. Such technologies promote health, performance, immunity, and pathogen control through a variety of mechanisms generally impacting the gastrointestinal tract and immune system [21,22,23,24,25]. The most commonly used products are often classified as prebiotics, probiotics, synbiotics, or postbiotics, based upon composition and functionality, though these definitions continue to evolve [26,27,28].

Original XPC™ (Diamond V, Cedar Rapids, IA, USA) is a postbiotic product consisting of functional metabolites produced as a proprietary *Saccharomyces cerevisiae* fermentation product (SCFP). As a newer category of products, postbiotics are generally considered preparations consisting of the bioactive compounds produced in controlled fermentation processes by specific microorganisms that ultimately confer a health benefit to the target host [29,30]. SCFP has demonstrated improvements in a variety of gut health, immunity, and production performance measures in commercial poultry, including lower corticosterone levels, heterophil/lymphocyte ratios, and physical asymmetry during stress events, reduced intestinal lesions and improved immune function during coccidia challenge, improved feed efficiency, growth, meat yield, and egg production, as well as foodborne pathogen reduction, including *Salmonella* [31,32,33,34,35,36,37,38,39]. In vitro gut fermentation models have associated SCFP with modulation of the microflora composition in a manner to synergistically reduce *Salmonella* Typhimurium and *Campylobacter jejuni* concentrations [40,41,42]. The purpose of this study was to evaluate dietary inclusion of SCFP, under real-world production conditions, on *Salmonella enterica* colonization, load, and overall burden in the litter and ceca of commercial broilers on a Honduran farm.

## 2. Materials and Methods

### 2.1. Experimental Design

A single commercial broiler farm was selected by the collaborating producer for inclusion into the study. The farm consisted of 12 open-sided houses with an average placement population of ~9400 birds per house. Houses were matched by feeder and drinker system type and insulation parameters to assign six houses each to two dietary treatments consisting of the standard industry diet (CON) or the standard industry diet supplemented with 1.25 kg/MT of postbiotic (SCFP). Prior to placement of the first flock, each house was fully cleaned and sanitized according to the company’s standard operating procedures, and new wood shavings or rice hull litter sourced and placed into each house with litter sampling prior to chick placement for the first rearing cycle. Litter was reutilized without amendment for subsequent rearing cycles except for top dressing in the brooding area at chick placements, wherein the same litter type was utilized with the exception of one house after the second rearing cycle. For each cycle, day of hatch Cobb 500 slow or Ross 308 slow broiler chicks sourced from the company’s suppliers were placed into each house and exposed to dietary treatments from Day 0 to the targeted market age of approximately 33–34 days. Sampling and laboratory personnel were blinded to treatment. Three consecutive flock cycles were evaluated in the study with houses remaining in the respective assigned treatment cohort.

### 2.2. Litter Sampling

Environmental sampling of the litter was conducted on four occasions to include sampling of the new litter prior to placement of the first flock cycle and at harvest of each cycle prior to placement of the subsequent flock. On each occasion and within each house, four litter swabs were collected. Briefly, for each house, new 1/2” × 9” nap paint rollers (Bates Choice Pro, Lafayette, LA, USA) were prehydrated with 200 mL of Buffered Peptone Water (BPW) (Symmag Suministros Industriales, San Pedro Sula, Honduras) and the roller device handle (Rubbermaid, Atlanta, GA, USA) utilized to furrow into the litter and roll the length of each house in a U-pattern in four replicates. Immediately upon sampling, each roller was placed into an appropriately labeled sterile sample bag (Twirl’Em^®^, LabPlas Inc., Montreal, QC, Canada) and placed on ice in a cooler for transport to the laboratory for analysis.

### 2.3. Ceca Sampling

For each flock and house within cycle at the target market age, 25 birds were randomly caught and removed from the house for sampling. Birds were humanely euthanized by cervical dislocation, dipped in a 400 ppm quaternary ammonium solution (Edwards Councilor, Virginia Beach, VA, USA), and prepared for necropsy. One cecal pouch was aseptically removed from each bird and placed into an appropriately labeled sterile sample bag and immediately placed on ice in a cooler for transport to the laboratory for analysis.

### 2.4. Salmonella Analysis

All samples were received into the laboratory and processed within 12–24 h of collection. Nap roller litter samples were prepared for analysis by the addition of 200 mL of BPW with subsequent hand agitation and rinsing for approximately 1–2 min, creating the primary sample. Each ceca sample was weighed, lacerated to expose contents, and 40 mL of BPW added to the tissue and contents with agitation for 1 min (Vortex Genie 2, USA Scientific, Ocala, FL, USA) to create a primary sample slurry. For both matrices, culture-based qualitative and quantitative *Salmonella* analyses were conducted in parallel. For detection of *Salmonella*, a 1 mL aliquot from each primary sample was removed and combined with 9 mL of BPW and incubated at 35° ± 2 °C for 18–24 h. After incubation, 0.5 ± 0.05 mL of the BPW enrichments were transferred into 10 mL Tetrathionate broth (TT) and 0.1 ± 0.02 mL into 10 mL Rappaport–Vassiliadis (RV) broth with incubation at 42°± 0.5 °C for 22–24 h. Tetrathionate and RV broths were then sampled with a 10 µL sterile loop and streak-plated for isolation of *Salmonella* on xylose lysine desoxycholate (XLD) and Rambach agar plates with incubation at 35° ± 2 °C for 18–24 h. Morphologically typical colonies were then confirmed as *Salmonella enterica* by colony-based *Salmonella* PCR (Biocontrol GDS, MilliporeSigma, Burlington, VT, USA) and an immunochromatographic lateral flow (Singlepath^®^ *Salmonella*, MilliporeSigma, Burlington, VT, USA). In parallel, three 1 mL aliquots of primary slurry were placed into the first three wells of a 96-well plasma tube plate and serially diluted in BPW to achieve a seven-dilution miniaturized most probable number assay (mMPN) according to Pavic et al. [43]. The sample mMPN positive well patterns were input into an MPN calculator to estimate MPN/mL of the primary sample which was then back-calculated to obtain MPN/swab and weight-adjusted MPN/g of ceca.

### 2.5. Statistical Analysis

Paired analytical outcomes reported by the laboratory for each sample were input into a spreadsheet by study variables including sample type, flock cycle, treatment code, and house. Data were analyzed in SAS Version 9.4 (SAS Institute, Cary, NC, USA) and R Version 4.1.0 (R Core Team, Vienna, Austria) with house as the experimental unit. The binomial response variable for the outcome of *Salmonella* prevalence (% positive) was determined for each individual sample when confirmation of a typical isolate was obtained from either the detection or mMPN assay or both. Litter swab *Salmonella* prevalence and load were modeled using a generalized linear mixed model and linear mixed model that were fit using the packages “lme4” [44] and “lmerTest” [45]. For prevalence, a binomial mixed-effects logistic regression was fit with litter type, treatment, their interaction, and a covariate for baseline prevalence at preplacement as fixed effects, and random effects for rearing cycle and pen. Rearing cycle was fit as a random effect because of the need to include additional covariates; as a result, the regression coefficients represent the average of the cycle-specific estimates. The interaction term between litter type and treatment was removed if *p* > 0.05. For litter swab *Salmonella* load, a linear mixed-effects model was fit with the same fixed and random effects, except the baseline prevalence covariate was replaced with a covariate for the *Salmonella* load measured at preplacement. Least-square/marginal means were estimated using the package “emmeans” [46]. Cecal prevalence data were modeled in PROC GLIMMIX using events/trials at the house level with fixed effects of treatment, cycle, and the interaction. Raw MPN/mL estimates were back-calculated by dilution volume to obtain MPN/swab and ceca adjusted by sample weight to obtain MPN/g prior to log_10_ transformation. Estimates of total *Salmonella* burden were calculated utilizing the cecal prevalence and load values obtained for each individual flock based on total birds at harvest. Cecal quantitative estimates were modeled in PROC MIXED with fixed effects of treatment, cycle, and the interaction. LS means estimates with pairwise differences for fixed effect of treatment were considered significant at *p* < 0.05. An observational and statistical outlier was removed from the SCFP cohort’s ceca samples in cycle 3 because of a sampling protocol deviation wherein the subsample of birds was removed and held overnight in a coop prior to sampling, which did not occur for any other house or cycle in the study. This was removed because this sampling deviation, in combination with feed and water withdrawal, may increase *Salmonella* in the sampled birds and thus be confounding to the estimate for the specific experimental unit [47,48,49].

## 3. Results

**Figure 1:**
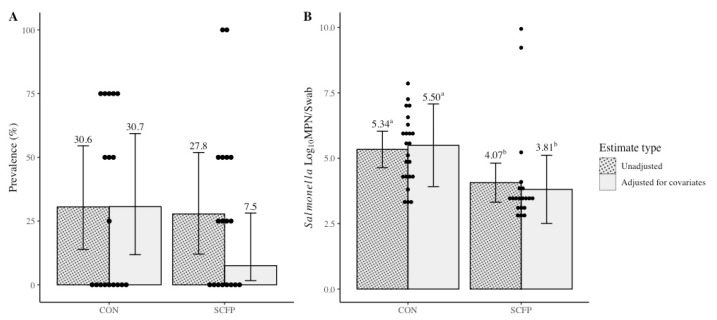
Treatment level *Salmonella enterica* litter prevalence (**A**) and load (**B**) LS means estimates with and without adjustment for covariates. Error bars reflect 95% confidence intervals with significant differences within estimate types denoted by superscripts ^a,b^ at *p* < 0.05. Dots represent individual observed values.

*Salmonella* litter prevalence across cycles of the trial were not different (*p* = 0.09) between CON (30.6%; adjusted 30.7%) or SCFP (27.8%; adjusted 7.5%), despite a lower observed and estimated prevalence for SCFP (Figure 1A). Preplacement litter swabs indicated variability of *Salmonella* presence in the fresh litter of some houses, and means were higher for those within the SCFP cohort (33.3%) as compared to CON (25.0%) (Table 1).

Cycle to cycle, observed litter prevalence was quite variable, with SCFP litter prevalence of 20.8%, 20.8%, and 41.7% for cycles 1, 2, and 3 and CON prevalence of 12.5%, 50.0%, and 33.3%, respectively (Table 1).

The difference in preplacement prevalence and litter types resulted in markedly different observed and adjusted mean estimates after controlling for their unbalanced distribution across treatment groups. Preplacement, mean *Salmonella* load in the culture-positive fresh litter samples was also observed to be higher in the houses assigned to the SCFP cohort (6.17 log_10_ MPN/swab) when compared to houses in the CON cohort (3.35 log_10_ MPN/swab), indicating that incoming litter or house contamination with *Salmonella* was not equivalent between cohorts (Table 1). Mean litter load across cycles in culture-positive houses after adjustment for litter type and preplacement load was different (*p* = 0.04) between CON (5.34 log_10_ MPN/swab; adjusted 5.50 log_10_ MPN/swab) and SCFP (4.07 log_10_ MPN/swab; adjusted 3.81 log_10_ MPN/swab), with *Salmonella* load in SCFP houses being lower by 1.69 log_10_ MPN/swab (95% CI: 0.107 to 3.26; Figure 1B). After each successive rearing cycle, observed mean litter loads in the SCFP cohort houses were 3.01, 3.54, and 4.85 log_10_ MPN/swab compared to CON houses at 5.77, 5.47, and 4.98 log_10_ MPN/swab, respectively (Table 1).

**Figure 2:**
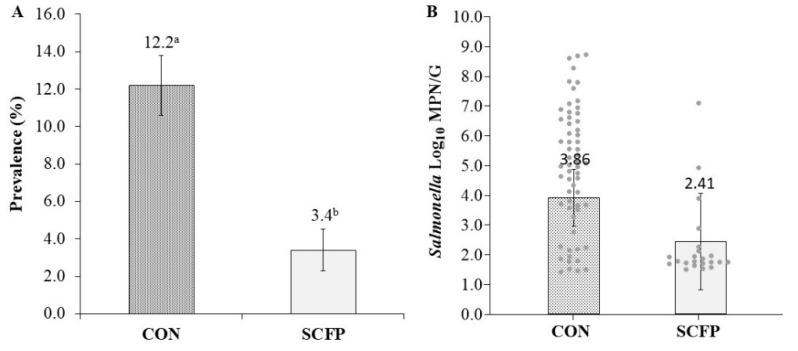
Treatment-level *Salmonella enterica* cecal prevalence (**A**) and load (**B**) LS means estimates. Error bars reflect prevalence estimate standard error and load estimate 95% confidence interval with significant differences denoted by superscripts ^a,b^ at *p* > 0.05.

Prevalence of *Salmonella* across all cycles within the ceca of birds from the SCFP cohort were significantly lower (3.4%; 95% CI: 1.8–6.4%) as compared to CON (12.2%; 95% CI: 9.2–15.9%), reflecting a 72.1% reduction (Figure 2A).

Within flock cycles, prevalence of *Salmonella* remained lower (*p* < 0.05) in SCFP cohort houses at 1.3%, 12.0%, and 2.4% as compared to CON houses at 7.3%, 22.0%, and 10.7% for cycles 1, 2, and 3, respectively (Table 2 and Table 3). Culture-positive *Salmonella* load for SCFP (2.41 log_10_ MPN/g; 95% CI: 0.81–4.01) was numerically (1.45 log_10_ MPN/g) lower (*p* = 0.121) than CON (3.86 log_10_ MPN/g; 95% CI: 2.91–4.81) (Figure 2B). Within flock cycles, SCFP cecal loads were 1.67, 1.95, and 3.62 log_10_ MPN/g as compared to CON loads at 2.98, 4.72, and 3.88 log_10_ MPN/g, reflecting no difference in cycle 1 (*p* = 0.53), a reduction (*p* = 0.016) of 2.77 for cycle 2, and no difference (*p* = 0.853) in cycle 3 (Table 3).

**Figure 3:**
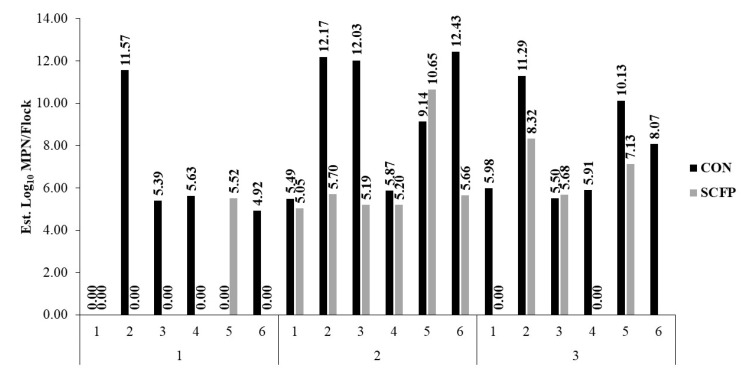
Estimated *Salmonella enterica* burden by flock within rearing cycle and treatment cohort. Values (MPN/flock) reflect livability-adjusted flock estimates obtained utilizing observed prevalence and load outcomes.

**Figure 4:**
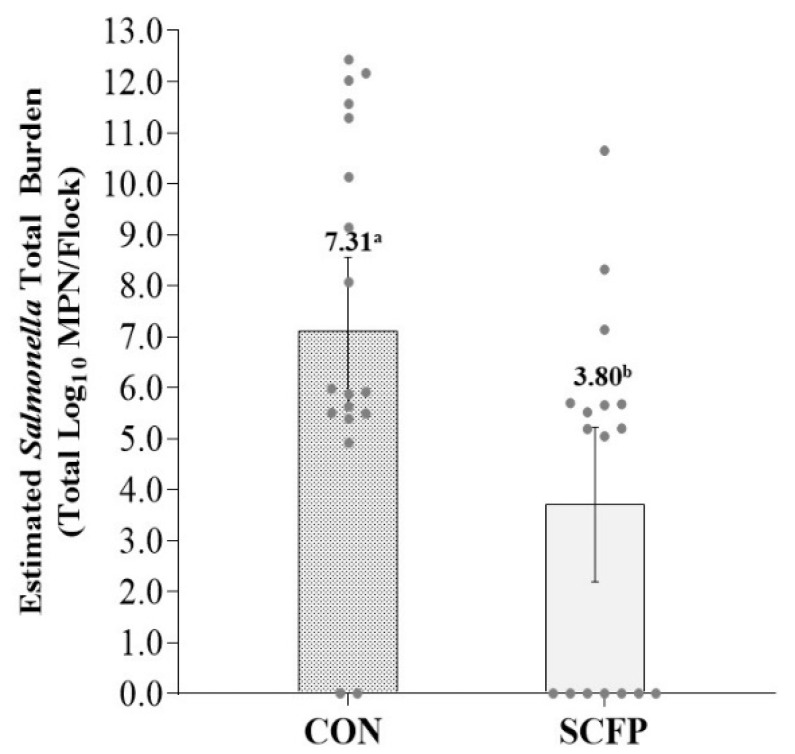
LS means estimated total *Salmonella enterica* burden by treatment. Error bars reflect 95% confidence interval with significant differences denoted by superscripts ^a,b^ at *p* < 0.05.

Estimates of total *Salmonella* burden going to harvest were 7.31 log_10_ MPN (95% CI: 5.80–8.82) and 3.80 log_10_ MPN (95% CI: 2.23–5.36) for CON and SCFP cohorts, respectively, a more than 1000-fold reduction (*p* = 0.0026) in cecal *Salmonella* total load associated with SCFP inclusion into the dietary ration (Figure 3 and Figure 4).

## 4. Discussion

*Salmonella enterica* are foodborne pathogens of significant public health risk globally in broiler meat and other poultry products. Feed-additive technologies that promote animal health and production while additionally conferring a preharvest food safety benefit may ultimately aid producers in their efforts to reduce pathogen risk when utilized in conjunction with a comprehensive food safety management plan. In this study conducted on a single Honduran commercial broiler farm, data support that broiler flocks fed SCFP had significantly reduced cecal prevalence and reduced loads of *Salmonella* when compared to same-farm flocks fed a typical industry diet without SCFP (CON). Additionally, these data present *Salmonella* prevalence information from within a representative commercial broiler operation in Honduras. To our knowledge, no such recent reports are available in the scientific literature.

In broiler and other poultry production, litter management practices, quality, and microbial composition are significant factors associated with pathogen status in flocks, particularly in regions where reuse may be a common practice. Litter microbiome analyses have demonstrated associations between the physicochemical characteristics of the litter, the abundance of specific taxa, the associated ability to isolate foodborne pathogens such as *Salmonella* and *Campylobacter*, and the litter influence on gut colonization [50,51,52]. Specifically, for *Salmonella*, Machado and Hagerman [53] reported decreasing odds for the probability of detecting *Salmonella* in litter prior to harvest with successive litter reuses up to six rearing cycles, after which, however, the odds began to increase. Conversely, in another study, *Salmonella* detections in recycled litter continued to decrease with up to 14 reuse cycles [54]. Other factors, such as house construction, pad composition, soil type, and litter type may also influence the ability of *Salmonella* to persist in litter [55]. The microbial ecology of litter is dynamic and complex with environmental conditions and management practices, potentially creating conditions favoring the persistence of *Salmonella* and other pathogens. These risks, however, likely vary greatly between regions, practices, and operations.

What may be more consistent is the cyclical influence between the litter and the host’s gut, thereby influenced by environmental and dietary treatments [56]. In this study, we observed that including SCFP in the diets of broilers did appear to affect a subtle shift in litter *Salmonella* status over the course of three consecutive reuses. Despite an observed higher mean prevalence and load in the fresh litter for the SCFP cohort of houses pre-placement, the model-adjusted across-cycle estimate indicated an overall lower *Salmonella* MPN/swab load for the SCFP cohort. The commercial farm utilized two different litter types in the houses’ preplacement of the first rearing cycle: specifically, rice hull or wood shavings. While comparing litter types was not an objective of the study, the effect of litter type was investigated utilizing general linear mixed models. For these models, the SCFP cohort demonstrated significantly less *Salmonella* loads in the litter regardless of type. Notably, litter type was not equally balanced between treatments. Sampling conducted pre-placement revealed two houses within each treatment testing positive, for which both litter types were represented. Considering all other houses in the trial were negative with both fresh litter types at preplacement, this observation may suggest house contamination (e.g., ineffective sanitation or cross-contamination from outside source into the specific barns) rather than a contaminated litter source. This observation highlights a key challenge and example of the limitations in conducting real-world research in commercial operations and may warrant future research needs to investigate *Salmonella* survival and proliferation as influenced by litter type on a commercial operation scale.

Regardless of origin, *Salmonella* in the rearing environment leads to host exposure and the chance for colonization of the gut. The epidemiological triad between the agent, environment, and host is a dynamic that contributes to the persistence of a pathogen such as *Salmonella* in the production and controlled rearing of broilers. Though not quantifiable in the present study, the observation of reduced litter load could contribute to a lower re-infection pressure and, therefore, decreased *Salmonella* cecal prevalence and loads observed within the SCFP cohort as compared to the CON cohort flocks reared on the respective litters over the course of the trial. In controlled research, *Salmonella* serotype status in contaminated litter has been associated with isolation in the crop and ceca, even within a short 12 h window of feed withdrawal and contaminated litter exposure [57]. Feed withdrawal prior to harvest has been demonstrated to significantly increase *Salmonella* detection in the crop and could lead to carcass contamination during harvesting [58]. Indeed, Berghaus and colleagues reported that *Salmonella* loads in environmental boot swabs and litter samples had the strongest association with loads of *Salmonella* in pre- and post-chill carcass rinses [59]. The risk of transfer from litter to organ colonization and subsequently carcass contamination during harvest is multifactorial and complex; nonetheless, it is likely to be influenced by the quantitative loads of *Salmonella* exposure. As such, the reduced *Salmonella* litter loads observed in the SCFP cohort of this study may be key in reducing individual exposure in subsequent flock placements and thereby contribute to overall long-term management. Future research could explore litter microbiome composition and *Salmonella* survival as specifically influenced by dietary treatments of birds.

While litter and other farm inputs (such as feed, water, and personnel) expose naïve individuals or populations to *Salmonella*, the ability of *Salmonella* to colonize and propagate within and between individuals in the population is largely influenced at the individual level by the host’s gut microbiome and immune system. Resistance to colonization by *Salmonella* may be partially attributed to short-chain volatile fatty acid (VFA) and other metabolite products from members of the microbiome, as well as maintenance of gut barrier function and immunity [60]. SCFPs have been shown to increased VFA concentrations for acetate and butyrate and promote favorable shifts in abundance of beneficial taxa and inhibition of *Salmonella* propagation during challenge [40,41,42,61]. In vivo research has identified immunomodulatory benefits of feeding SCFP to broilers, including those challenged with *Eimeria tenella,* though no *Salmonella*-specific mechanisms have been investigated to date [36,37]. These reports suggest that SCFP may impart an anti-*Salmonella* effect through multiple pathways and modes of action, though more research is warranted to determine anti-*Salmonella*-specific mechanisms.

Controlled research evaluating the effects of SCFP to reduce *Salmonella* Enteritidis challenges has demonstrated quantitative reductions in cecal contents [62,63]; however, reductions in prevalence (% positive) for direct challenge studies are commonly not observed due to extremely high challenge doses administered via oral gavage. Real-world industry conditions have exposure routes and doses which vary greatly [59]. When combined with stress, disease challenge, and other factors of commercial production not easily replicated in controlled research settings, the efficacy of SCFP and similar products may become more evident. This study demonstrated that SCFP reduced *Salmonella* prevalence compared to control flocks, and prevalence within SCFP flocks were consistently lower within and between flock-rearing cycles. The consistent reduction in the number of individuals positive for *Salmonella* was also coupled with those individuals and the populations, on average, having significantly lower cecal *Salmonella* loads. Notably, the combined reductions observed cycle over cycle in both cecal *Salmonella* prevalence and loads in populations of the SCFP cohort indicate that there would be fewer individuals in a flock shedding *Salmonella* at lower loads back into the litter during rearing and thus likely influencing the subsequent flock placement. While our trial only observed three consecutive cycles, the data do appear to directionally support this concept of *Salmonella* transmission, and future work may consider following an increased number of cycles. If this is the case, products such as SCFP may impart a compounding benefit over time, particularly in regions where litter reuse and litter amendment interventions are commonly utilized.

Ultimately, efforts to reduce *Salmonella* risk rely on multiple interventions strategically utilized throughout the processing continuum. At harvest and in processing, several physical and chemical interventions, often validated for multi-log reductions of indicator organisms or pathogens such as *Salmonella*, are commonly employed to reduce microbial contamination. These include, but are not limited to, hot water, steam, chlorine, organic acids, and others [64,65]. The efficacy of these interventions may, therefore, be impacted by the incoming microbial burden that must be mitigated. Utilizing the *Salmonella* estimates obtained for each flock on trial in combination with placement numbers adjusted for mortality, we estimated the total burden of *Salmonella* to processing. While sufficient evidence is lacking to directly correlate cecal prevalence and loads to finished product contamination risk, litter loads have been associated with carcass contamination, and thus these relationships at the flock-level may be useful, in the very least, as estimates of incoming *Salmonella* burden to be managed. Flock-level burden estimates varied greatly across cycles and within treatment cohorts. Notably, fewer overall flocks within the SCFP cohort (*n* = 4) had a load burden exceeding 6.00 log_10_ MPN as compared to the CON cohort which had eight flocks, of which six exceeded 10.00 log_10_ MPN (Figure 3). From a practical point of view, higher cumulative burden at a processing plant could constrain efficacy of postharvest processing controls and the food safety management system. Products such as SCFPs may be beneficial towards reducing the overall load burden at these facilities, and future research may warrant sampling of postharvest matrices.

*Salmonella* prevalence and serovar distribution in food animal production are recognized as being both geographically diverse and influenced by seasonality [66,67,68,69]. Therefore, limitations to this study may include the geographical isolation of a single farm in Honduras studied over an approximate 4-month window. However, a critical benefit in this study was the replication of dietary treatments within the single farm in parallel, which is often not feasible for many farm operations. Serotyping or molecular typing of isolates may have yielded additional insights into this study. However, serotyping in commercial farms is not always approved by stakeholders, thus another general limitation of the study. Lastly, as previously noted, global diversity in soil type, pad construction, litter types, house types, and farm management practices exist, all of which may influence *Salmonella* ecology uniquely and thus warrant continued evaluation of SCFP postbiotic.

Together, these data demonstrate the association and influence that dietary inclusion of SCFP imparts on multi-flock qualitative and quantitative *Salmonella* reductions. These reductions, therefore, may lead to an overall lower *Salmonella* burden on farms and are likely to challenge a processing facility. SCFP postbiotic demonstrates potential to serve as an effective preharvest intervention, thus contributing to a multi-hurdle farm-to-fork *Salmonella* food safety program.

## Figures and Tables

**Table 1:** Observed prevalence and load of *Salmonella enterica* in the litter of broiler houses freshly sourced and after each of three consecutive rearing cycles of birds fed either CON or SCFP diets.

Variable	Treatment	Preplacement	Cycle 1	Cycle 2	Cycle 3
*Salmonella* Prevalence (%; no. positive/total no. samples)	CON	25.0 (6/24)	12.5 (3/24)	50.0 (12/24)	33.3 (8/24)
	SCFP	33.3 (8/24)	20.8 (5/24)	20.8 (5/24)	41.7 (10/24)
^a^*Salmonella* Concentration (log_10_ MPN/swab of culture-positive samples)	CON	3.35 (0.66)	5.77 (1.85)	5.47 (1.48)	4.98 (0.94)
	SCFP	6.17 (0.99)	3.01 (0.32)	3.54 (0.45)	4.85 (2.56)

^a^ Observed MPN/swab mean within-cycle load and standard deviation.

**Table 2:** Observed prevalence and load of *Salmonella enterica* in the ceca of broilers at market age fed either CON or SCFP diets by flock-rearing cycle and estimated treatment level within-cycle total burden.

		Flock-Rearing Cycle		
Variable	Treatment	Cycle 1	Cycle 2	Cycle 3
*Salmonella* Prevalence (%; no. positive/total no. samples)	CON	7.3 (11/150)	22.0 (33/150)	10.7 (16/150)
	SCFP	1.3 (2/150)	12.0 (18/150)	2.4 (3/125)
^a^*Salmonella* Concentration (log_10_ MPN/g of culture-positive samples)	CON	4.12 (2.58)	5.44 (1.84)	4.33 (1.94)
	SCFP	1.67 (0.04)	2.26 (1.34)	3.62 (1.33)

^a^ Observed MPN/g mean within-cycle load and standard deviation.

**Table 3:** LS means estimates for prevalence and load of *Salmonella enterica* in the ceca of broilers at market age fed either CON or SCFP diets by flock-rearing cycle and estimated treatment level within-cycle total burden.

		Flock-Rearing Cycle		
Variable	Treatment	Cycle 1	Cycle 2	Cycle 3
* Salmonella Prevalence (%; no. positive/total no. samples)	CON	7.3 ± 2.1 ^a^ (11/150)	22.0 ± 3.4 ^a^ (33/150)	10.7 ± 2.5 ^a^ (16/150)
	SCFP	1.3 ± 0.9 ^b^ (2/150)	12.0 ± 2.7 ^b^ (18/150)	2.4 ± 1.4 ^b^ (3/125)
* Salmonella Cecal Concentration (log_10_ MPN/g of culture-positive samples)	CON	2.98 ± 0.91	4.72 ± 0.71 ^a^	3.88 ± 0.74
	SCFP	1.67 ± 1.82	1.95 ± 0.77 ^b^	3.62 ± 1.18
*Salmonella* Est. Cecal Burden to Plant (log_10_ MPN)	CON	4.59 ± 1.28	9.52 ± 1.28	7.81 ± 1.28
	SCFP	0.92 ± 1.28	6.24 ±1.28	4.23 ± 1.40

Treatment LS mean estimates and SEM for within-flock cycle. * Superscripts ^a,b^ denote significant treatment differences within-cycle at *p* < 0.05.

## Data Availability

Observed microbiological data is reported within the article. Restrictions apply to the availability of additional information due to 3rd party proprietary information associated with collaborating producer.
